# Neutrophil Extracellular Traps Promote Aberrant Macrophages Activation in Behçet’s Disease

**DOI:** 10.3389/fimmu.2020.590622

**Published:** 2021-02-05

**Authors:** Lu Li, Xin Yu, Jinjing Liu, Zhimian Wang, Chaoran Li, Jing Shi, Luxi Sun, Yi Liu, Fengchun Zhang, Hua Chen, Wenjie Zheng

**Affiliations:** ^1^ Department of Rheumatology, Peking Union Medical College Hospital, Chinese Academy of Medical Sciences, Peking Union Medical College, The Ministry of Education Key Laboratory, National Clinical Research Center for Dermatologic and Immunologic Diseases, Beijing, China; ^2^ School of Nursing, Peking Union Medical College, Beijing, China; ^3^ Department of Rheumatology and Immunology, Rare Diseases Center, West China Hospital, Sichuan University, Chengdu, China

**Keywords:** neutrophil extracellular traps, macrophages, oxidized DNA, histone H4, Behçet’s disease

## Abstract

Neutrophil extracellular traps (NETs) are upregulated and promote thrombosis in Behçet’s disease (BD). However, whether NETs promote autoinflammation in BD remains unclear. This study aimed to investigate the potential role of NETs in promoting macrophage activation in BD. Firstly, we quantified NETs by measuring double-stranded DNA (dsDNA) using PicoGreen and calculating the proportion of NETosis. Then macrophages were stimulated with BD- or healthy controls (HC)-derived NETs, and IL-8 and TNF-α production and IFN-γ^+^ CD4^+^ T cells differentiation were measured using ELISA and flow cytometry, respectively. The protein components in NETs were analyzed by western blot. Macrophages were stimulated with Histone H4 neutralized NETs, and IL-8 and TNF-α production were measured using ELISA. The level of 8-hydroxydeoxyguanosine (8-OHdG) DNA in NETs was measured using ELISA. The levels of reactive oxygen species (ROS) in serum and neutrophils were measured using ROS probes by a microplate reader and flow cytometry. We found that circulating NETs and neutrophil-derived NETs were significantly higher in BD than HC. BD NETs stimulated macrophages produced higher levels of IL-8 and TNF-α, and promoted IFN-γ^+^ CD4^+^ T cells differentiation. BD NETs were enriched in Histone H4, and neutralizing Histone H4 abrogated the BD NETs-mediated IL-8 production by macrophages, but not TNF-α. Also, BD neutrophils produced more 8-OHdG DNA than HC neutrophils, and the percentage of 8-OHdG DNA in dsDNA from BD neutrophils was also higher than that of HC neutrophils. The ROS levels in serum and neutrophils were both higher in BD than HC. Our findings suggested that excessive BD NETs promoted macrophages activation and facilitated IFN-γ^+^ CD4^+^ T cells differentiation. Higher levels of Histone H4 and oxidized DNA in BD NETs might mediate macrophages hyperactivation.

## Introduction

Behçet’s disease (BD) is a chronic inflammatory disease characterized with neutrophils hyperactivation (1). Beyond chemotaxis, phagocytosis, and superoxide production, neutrophils release neutrophil extracellular traps (NETs), the extracellular web-like structures of decondensed chromatin decorated with proteins from the cytosol and neutrophil granules (2). NETs are enriched in serum and deposit in inflamed vessels, cutaneous vasculitis and panniculitis in BD patients (3, 4), which are cytotoxic for endothelial cells (4) and promote procoagulant state (3) in BD patients.

NETs are internalized and degraded by macrophages (5) and promote the release of proinflammatory cytokines (6, 7). NETs are the primary source of extracellular DNA and induce macrophages activation in Adult-onset Still’s disease (8). However, it remains unclear whether NETs contribute to the inflammatory milieu in BD, such as regulating macrophage activation and imbalanced Th1 cells polarization.

In this study, we aimed to investigate whether NETs promoted macrophages activation in BD and its underlying mechanisms.

## Methods

### Patients and Controls

Treatment-naive BD patients (n = 38) fulfilled the international criteria for Behçet’s disease (ICBD) were enrolled in Peking Union Medical College Hospital (PUMCH) from April 2018 to November 2020. The following clinical data were collected: clinical manifestations, erythrocyte sedimentation rate (ESR), C reaction protein (CRP), and BD Current Activity Form 2006 (BDCAF2006) (**Supplementary Table**). Age- and sex-matched healthy volunteers (n = 36) without a history of autoimmune diseases were enrolled as healthy controls (HC). The study was approved by the institutional ethics review board of PUMCH. All participants provided written informed consent.

### Neutrophils Isolation and Serum Collection

Peripheral blood samples were collected from BD and HC. Neutrophils were isolated using Ficoll density gradient centrifugation according to the manufacturer’s instructions at 1,800× rpm/min for 20 min at 24°C. The neutrophil layer was harvested, treated with erythrocyte lysis buffer, washed with PBS, and resuspended in RPMI-1640 medium. HC and BD serum was prepared from serum collection tubes and collected after centrifugation at 3,500×rpm/min for 5 min and stored at −80°C until analysis.

### Double-Stranded DNA (dsDNA) Quantification

HC and BD neutrophils (1×10^6^) were unstimulated or stimulated with 25 nM PMA (Sigma, USA) for 4 h at 37°C, 5% CO_2_ in 24-well plates. The supernatant was centrifuged at 500×g for 10 min and was stored at −80°C until use. The supernatant and HC and BD serum were 1:10 diluted and quantified using Quant-iT PicoGreen dsDNA assay kit (Invitrogen, USA). The samples were incubated with PicoGreen 5 min at room temperature and protected from light. The plates were then excited at 485 nm, and the fluorescence emission intensity was measured at 520 nm.

### Immunofluorescence Staining of NETs

Neutrophils (5 × 10^5^) were seeded onto poly-L-Lysine-coated coverslips in 24-well plates and were unstimulated or stimulated with 25 nM PMA for 4 h at 37°C, 5% CO_2_. Cells were fixed with 4% paraformaldehyde for 10 min, then were washed with PBS, permeabilized with 0.5% Triton X-100 for 10 min and blocked with 2% BSA for 30 min. Cells were incubated with rabbit anti-neutrophil elastase (NE) antibody (1:500; Abcam, UK, ab131260) overnight at 4°C, washed with PBS, and incubated with Alexa 488-conjugated goat anti-rabbit antibody (Invitrogen, USA, A11034) for 1 h, then incubated with DAPI (Beyotime, China) for 5 min at room temperature. The washed coverslips were mounted onto slides and were imaged using confocal microscopy (Nikon microscope, Nikon NIS Elements v 4.20 software; Japan). The proportion of NETs formation (NETosis) induced in neutrophils was quantified using Image J software. The percent of NETosis was calculated as follows: (Number of cells showing NETosis/Total number of cells) × 100%.

### NETs Production

Neutrophils (2×10^6^) were stimulated with 25 nM PMA for 4 h at 37°C, 5% CO_2_ in 6-well plates to induce NETs formation. The supernatant was discarded and wells were washed with ice-cold DPBS. The washed solution was centrifuged at 400×g for 10 min at 4°C. Neutrophils and cell debris were pelleted at the bottom, leaving the supernatants enriched with NETs. Then NETs were pelleted at 17000×g for 10 min at 4°C. Pellets containing NETs were resuspended together in cold PBS and stored at −80°C until use. The DNA concentrations of NETs were measured using a spectrophotometer (9).

### NETs Stimulated Macrophages

Monocytes were isolated from HC peripheral blood mononuclear cells (PBMC) using CD14 magnetic beads (Miltenyi, Germany) and were incubated in DMEM supplemented with 10 ng/ml M-CSF (Sigma, USA) and 10% FBS for 7 days to differentiate to human monocyte-derived macrophages (HMDM). HMDM were stimulated with NETs (10 μg) from PMA-stimulated HC and BD neutrophils for 24 h. Supernatant IL-8 and TNF-α were measured by ELISA (Biolegend, USA). Furthermore, naive CD4^+^ T cells (1×10^5^) were isolated from HC PBMCs using naive CD4^+^ T magnetic beads (Miltenyi, Germany) and were incubated with HMDM (2×10^4^) stimulated with plate-bound anti-CD3, soluble anti-CD28, IL-2, and anti-IL-4 for 5 days. Cells were stimulated with a leukocyte activation cocktail (Bioscience, USA) for 5 h, and IFN-γ^+^ CD4^+^ (Biolegend, USA) T cells were measured by a flow cytometer.

### Immunoblotting of NETs Proteins

NETs from PMA-stimulated HC and BD neutrophils with equal DNA quantity were digested with 20 U/ml DNaseI (Sigma, USA) for 30 min at 37°C and stopped with 5 mM EDTA. Proteins were precipitated with cold acetone at −20°C overnight and pelleted at 14,000×g for 10 min at 4°C. The pellets were resuspended in lysis buffer containing 1% Triton X-100, 150 mM NaCl, 20 mM HEPES and protease inhibitors (Beyotime, China). Proteins were loaded with SDS loading buffer, denatured at 95°C for 5 min, and stored at −80°C. NETs proteins were loaded onto 4%–20% polyacrylamide gels, and the gels were transferred to PVDF membranes for 1 h at 200 mA. Membranes were blocked with QuickBlock blocking buffer (Beyotime, China) and were incubated with anti-Histone H1, anti-Histone H2A, anti-Histone H2B, anti-Histone H3, anti-Histone H4, anti-NE, anti-S100A8 (rabbit, Abcam, UK) antibody at 4°C overnight. HRP-conjugated goat anti-rabbit antibodies (Abcam, UK) were incubated for 1 h. Blots were developed with chemiluminescence and detected by Tanon 5200 (China). Protein levels were quantified by Image J software and measured with absolute optical density values, given no reference control protein available.

### Histone H4 Neutralization

NETs (10 μg) from PMA-stimulated HC and BD neutrophils were digested with 20 U/ml DNaseI (Sigma, USA) for 30 min, and NETs and Histone H4 were pretreated with 1:50 anti-Histone H4 antibody (Cell signaling, USA) for 1 h. Macrophages were stimulated with anti-Histone H4-treated or untreated Histone H4 (20 μg, Biolabs, USA), BD NETs or HC NETs for 24 h. Supernatant IL-8 and TNF-α were measured by ELISA (Biolegend, USA) at 450 and 570 nm.

### 8-hydroxydeoxyguanosine (8-OHdG) DNA Quantification

Oxidized DNA in HC and BD serum and neutrophils culture supernatant were measured using 8-OHdG ELISA Kit (CUSABIO, China), according to the manufacturer’s instructions. The samples were incubated with diluted 8-OHdG antibody 30 min at 37°C, followed by washing three times wells and incubating with HRP-conjugated streptavidin 30 min at 37°C. The assay was developed with TMB, and absorbance was analyzed at 450 nm.

### Reactive Oxygen Species (ROS) Detection

HC and BD neutrophils (1×10^6^) were unstimulated or stimulated with 25 nM PMA (Sigma, USA) for 10 min at 37°C, 5% CO_2_ in 24-well plates. Then neutrophils were incubated with ROS probe (DCFH-DA, Solarbio, China), and median fluorescence intensity (MFI) was measured by flow cytometry. HC and BD serum ROS were detected by BBoxiProbe O12 assay kit (BestBio, China). HC and BD serum were incubated with O12 probe 15 min at 37°C and protected from light. The samples were excited at 488 nm, and the fluorescence emission intensity was measured at 530 nm.

### Statistical Analysis

Data were expressed as mean ± Standard Deviation or median (range). The Kolmogorov-Smirnov test tested the normality of the distribution of data. Comparisons between two groups were performed using Student’s t-test or Mann-Whitney U test, and a paired t-test was used to compare the differences between before and after treatment. Multiple comparisons were performed by ANOVA with LSD for *post hoc* test if the data is normal distribution and homogeneity of variance. Correlations were calculated using Pearson correlation analysis. A two-tailed p-value < 0.05 was considered statistically significant. All statistical analysis was performed using SPSS V.22.0 software (IBM, USA).

## Results

### BD Neutrophils Produced a Higher Level of NETs

**Figure 1:**
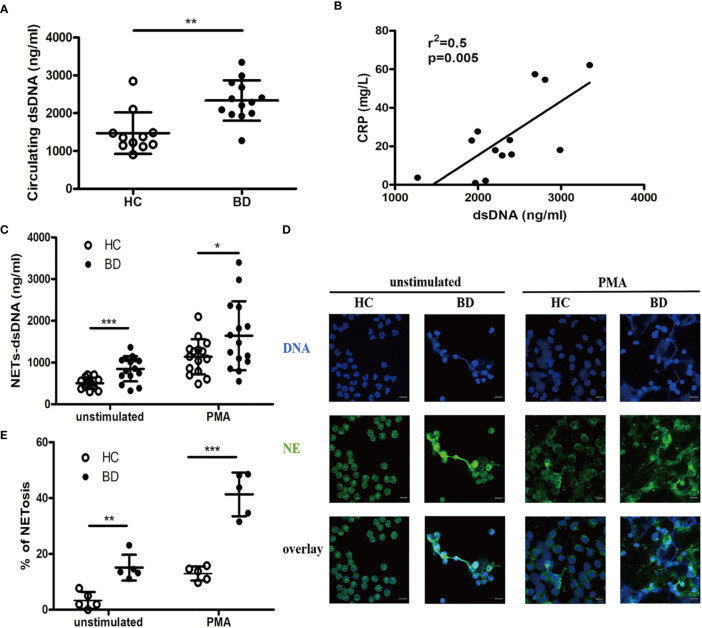
BD neutrophils produce higher level of neutrophil extracellular traps (NETs). **(A)** Serum dsDNA from HC (n = 11) and BD (n = 13). **(B)** Correlation of serum dsDNA with CRP in BD (n = 13). **(C)** Unstimulated or PMA-stimulated HC (n = 15) and BD (n = 15) neutrophils were incubated for 4 h, and supernatant dsDNA was measured using PicoGreen. **(D)** Representative immunofluorescence staining of NETs produced by unstimulated or PMA-stimulated HC and BD neutrophils. Green and blue denote neutrophil elastase (NE) and DNA, respectively. magnification 400×, zoom in×3, scale bars = 10 µm. **(E)** The proportion of NETosis induced in unstimulated and PMA-stimulated HC (n = 5) and BD (n = 5) neutrophils. Bars indicate mean value and Standard Deviation. Mann-Whitney U test **(A)**, Pearson correlation analysis **(B)** and Student’s t-test **(C, E)** were applied. *p < 0.05, **p < 0.01, ***p < 0.001. BD, Behçet’s disease; HC, healthy controls; ns, not significant.

We first measured circulating NETs in BD patients and HC. The serum dsDNA level was significantly higher in BD patients than that in HC [2290 (1272, 3345) ng/ml vs. 1355 (904.1, 2847) ng/ml, p = 0.0031] (**Figure 1A**), which was positively correlated with CRP (r^2^ = 0.5, p = 0.005) (**Figure 1B**). Furthermore, both unstimulated (849 ± 302.3 ng/ml vs. 503.8 ± 130.5 ng/ml, p = 0.0004) and PMA-stimulated (1,641 ± 827.4 ng/ml vs. 1,137 ± 419.8 ng/ml, p = 0.0443) neutrophils from BD patients produced more dsDNA than those from HC (**Figure 1C**). Immunofluorescence staining revealed that both unstimulated (15.1 ± 4.6% vs. 3.3 ± 3%, p = 0.0015) and PMA stimulated (41.3 ± 7.8% vs. 12.9 ± 2.5%, p < 0.0001) neutrophils from BD patients produced significantly more NETs than HC neutrophils, showing extensive NETs formation characterized with neutrophils swollen, changed in shape and spontaneously releasing net-like structure containing NE and DNA (**Figures 1D, E**). Together, neutrophils from BD produced more NETs than those from HC.

### BD NETs Promoted Macrophages Activation

**Figure 2:**
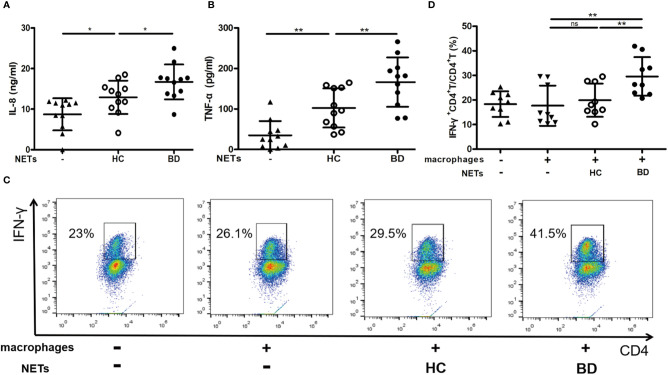
BD neutrophil extracellular traps (NETs) promote macrophages activation. Macrophages were stimulated with HC NETs (n = 11) or BD NETs (n = 11) for 24 h, and **(A)** IL-8 and **(B)** TNF-α production were measured using ELISA. NETs-stimulated or untreated macrophages were incubated with naive CD4^+^ T cells for 5 days, and **(C, D)** IFN-γ^+^ CD4^+^ T cells (n = 9) were measured by flow cytometry. Bars indicate mean value and Standard Deviation. One-way ANOVA with LSD for post-hoc test was applied. *p < 0.05, **p < 0.01. BD, Behçet’s disease; HC, healthy controls. ns, not significant.

We then examined whether NETs promoted macrophages activation in BD. We stimulated HC macrophages with the equal DNA quantity of NETs and found that BD NETs significantly induced macrophages to produce IL-8 (16.7 ± 4.3 ng/ml vs. 12.9 ± 4.1 ng/ml, p = 0.04) and TNF-α (166.4 ± 61.1 pg/ml vs. 102.4 ± 48.4 pg/ml, p = 0.005) compared with HC NETs (**Figures 2A, B**). We further incubated NETs-stimulated macrophages with naive CD4^+^ T cells and observed that BD NETs-stimulated macrophages promoted more IFN-γ^+^ CD4^+^ T cells differentiation than HC NETs-stimulated macrophages (29.6 ± 7.8% vs. 19.9 ± 6.8%, p = 0.007) (**Figures 2C, D**). Therefore, BD NETs promoted macrophages to produce more proinflammatory cytokines and facilitated Th1 cells differentiation, suggesting that NETs played a role in macrophages activation in BD.

### Elevated Histone H4 in BD NETs Promoted Macrophages to Overproduce IL-8

**Figure 3:**
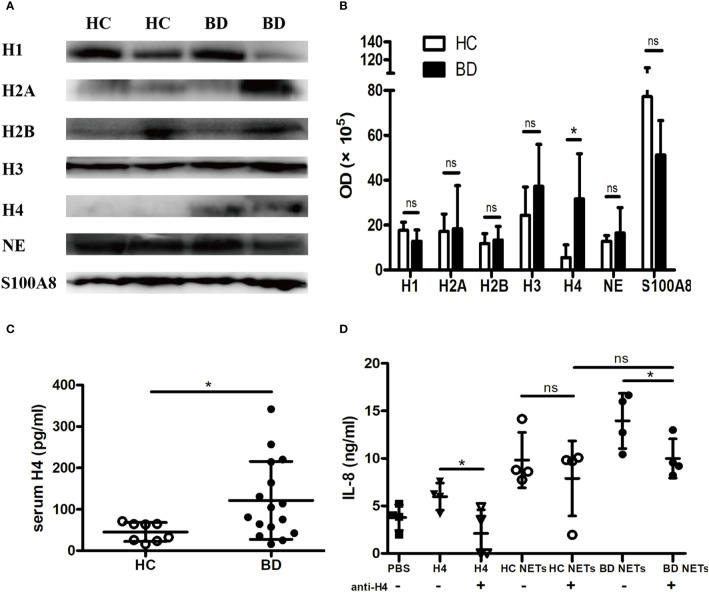
Histone H4 in BD neutrophil extracellular traps (NETs) promotes macrophages activation. **(A)** Representative images and **(B)** summary of western blot of Histone H1, Histone H2A, Histone H2B, Histone H3, Histone H4, NE, and S100A8 in HC NETs (n = 4) and BD NETs (n = 4). **(C)** Serum Histone H4 from HC (n = 8) and BD (n = 16) were measured by ELISA. **(D)** HC NETs (n = 4) or BD NETs (n = 4) were pretreated with anti-Histone H4 antibody, and stimulated macrophages for 24 h. IL-8 production was measured using ELISA. Bars indicate mean value and Standard Deviation. Mann-Whitney U test (B-C), paired t-test **(D)** and Student’s t-test **(D)** were applied. *p < 0.05. BD, Behçet’s disease; HC healthy controls; ns, not significant.

We further elucidated the mechanism of the proinflammatory effect of BD NETs. Given histones are the backbone proteins of DNA and are essential components of NETs. We first investigated the main protein composition in BD NETs. We measured Histone H1, Histone H2A, Histone H2B, Histone H3, Histone H4, as well as NE and S100A8, the major non-histone proteins of NETs. We observed that BD NETs were enriched in Histone H4 [OD, 289,076 (144,365, 544,038) vs. 42,121 (6,958, 129,625], p = 0.0286) (**Figures 3A, B**). Additionally, we found that serum Histone H4 level was significantly higher in BD patients than HC [92.6 (15.9, 341.9) pg/ml vs. 48.1 (15.8, 71.4) pg/ml, p = 0.03] (**Figure 3C**). To confirm whether Histone H4 promoted macrophage activation, we neutralized Histone H4 in NETs and evaluated IL-8 and TNF-α production by macrophages. Neutralizing Histone H4 abrogated the BD NETs-mediated IL-8 overproduction by macrophages (14 ± 2.9 ng/ml vs. 10 ± 2.1 ng/ml, p = 0.02), but not HC NETs (**Figure 3D**). In contrast, there was no difference in TNF-α production before and after neutralizing Histone H4 both in HC and BD NETs (**Supplementary Figure 1**), suggesting Histone H4 might promote the overproduction of IL-8 but not TNF-α. In summary, BD NETs abnormally contained a higher level of Histone H4, and elevated Histone H4 in BD NETs might contribute to promote macrophages activation to overproduce IL-8.

### BD NETs Contained More Oxidized DNA

**Figure 4:**
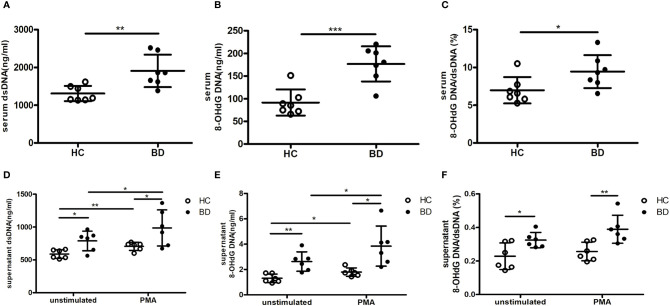
BD neutrophil extracellular traps (NETs) contain more oxidized DNA. Serum **(A)** dsDNA and **(B)** 8-OHdG DNA from HC (n = 7) and BD (n = 7). **(C)** The percentage of 8-OHdG DNA in dsDNA from HC (n = 7) and BD (n = 7) serum. Supernatant **(D)** dsDNA and **(E)** 8-OHdG DNA produced by unstimulated and PMA-stimulated HC neutrophils (n = 6) and BD neutrophils (n = 6). **(F)** The percentage of 8-OHdG DNA in dsDNA from unstimulated and PMA-stimulated HC neutrophils (n = 6) and BD neutrophils (n = 6). Bars indicate mean value and Standard Deviation. Student’s t-test **(A–F)** and paired t-test **(D, E)** were applied. *p < 0.05, **p < 0.01, ***p < 0.001. BD, Behçet’s disease; HC, healthy controls; ns, not significant.

We also explored the abnormal DNA composition in BD NETs. Given that oxidized DNA of NETs was reported as a potent inducer of proinflammatory cytokines (10), we measured the percentage of 8-OHdG DNA, an oxidized DNA, in dsDNA in BD NETs and HC NETs. Consistently with prior result, the serum dsDNA level was higher in BD (**Figure 4A**). As expected, the quantity of 8-OHdG DNA and the percentage of 8-OHdG DNA in dsDNA were higher in BD serum than HC serum (176.8 ± 38.9 ng/ml vs. 91.6 ± 29 ng/ml, p = 0.0006; 9.4 ± 2.2% vs. 7 ± 1.7%, p = 0.0375, respectively) (**Figures 4B, C**). The supernatant dsDNA level was higher in unstimulated and stimulated BD neutrophils than that of HC neutrophils (**Figure 4D**). Furthermore, we measured supernatant 8-OHdG DNA from BD and HC neutrophils. Consistently, both unstimulated and stimulated BD neutrophils produced more 8-OHdG DNA than HC neutrophils (2.6 ± 0.8 ng/ml vs. 1.3 ± 0.3 ng/ml, p = 0.0032; 3.8 ± 1.6 ng/ml vs. 1.8 ± 0.3 ng/ml, p = 0.0111, respectively) (**Figure 4E**), and the percentage of 8-OHdG DNA in dsDNA from BD neutrophils were also higher than that of HC neutrophils, (0.3 ± 0.1% vs. 0.2 ± 0.1%, p = 0.0281; 0.4 ± 0.1% vs. 0.3 ± 0.1%, p = 0.0088, respectively) (**Figure 4F**). Thus, these results indicated that BD NETs were enriched in oxidized DNA. Because the oxidized DNA was induced by ROS, we further compared the ROS levels in serum and neutrophils isolated from HC and BD. The serum ROS level was higher in BD patients than that of HC (OD, 8.4 ± 4.7 vs. 5.6 ± 3.4, p = 0.0288) (**Supplementary Figure 2A**), and BD neutrophils produced more ROS than HC neutrophils both in resting (564.3 ± 98.5 MFI vs. 464.3 ± 59.3 MFI, p = 0.013) and PMA-stimulation conditions (1,311 ± 265.1 MFI vs. 1,000 ± 129.1 MFI, p = 0.0037) (**Supplementary Figure 2B**). Therefore, these data suggest the overproduction of ROS in BD neutrophils, which potentially induced the more oxidized DNA in BD NETs and then the higher proinflammatory macrophages’ response.

## Discussion

In this study, we confirmed that NETs were overproduced in BD. Furthermore, we demonstrated that BD NETs induced aberrant macrophages activation and the underlying mechanisms, which might be mediated by the higher levels of Histone H4 and oxidized DNA.

We confirmed that BD neutrophils produced more NETs than HC neutrophils, which indicated that BD neutrophils were overactivated. The underlying mechanism remains largely unknown. Sandro et al. show that a higher level of plasma sCD40L promotes excessive oxidative burst of neutrophils and the production of NETs in BD (11). In addition, elevated proinflammatory cytokines, chemokines and adhesion molecules in BD, such as IL-17, IL-8, MIP-2, CD54, and CD62E (12, 13), also regulate neutrophils activation, which might be the potential mechanism of NETs overproduction in BD.

NETs induce macrophages to produce proinflammatory cytokines in pyogenic arthritis, pyoderma gangrenosum and acne syndrome (6), and systemic lupus erythematosus (7). Given that BD is an autoinflammatory disease, we hypothesized that NETs from BD patients could also activate macrophages. We observed BD NETs promoted macrophages to produce IL-8 and TNF-α, the key proinflammatory cytokines in BD. IL-8 attracts, activates neutrophils and enhances neutrophils adhesion to endothelial cells (14), resulting in NETs-driven vicious inflammatory cascade response and driving the progression in BD. TNF-α is highly expressed in BD (15), and TNF-α blockades are effective for severe and refractory BD (16, 17). Furthermore, macrophages also regulate T cell activation. We found that macrophages stimulated with BD NETs promoted Th1 cells differentiation, a major player in BD (18). Therefore, our data highlighted that BD NETs might participate in macrophages overactivation in BD, and targeting NETs might be an approach to suppress Th1 cells differentiation in BD.

Over 164 proteins are reported in NETs (19), including histones, NE, calprotectin and other antimicrobial proteins (20, 21). Histones are the most abundant proteins in NETs, accounting for 70% of NET-associated proteins (20). Extracellular histones are potential mediators of systemic inflammatory diseases *via* activating TLR2, TLR4, TLR9 and NLRP3 inflammasomes, promoting cell-mediated apoptosis (22). Beyond histones, NE and S100A8, the major non-histone proteins in NETs are also agonists of TLRs and cytotoxic mediators (23, 24). However, no difference in NE or S100A8 was observed. We found that the higher level of Histone H4 was partially responsible for the proinflammatory effect of BD NETs. Histone H4 participates in tissue damage and organ injury by activating the MAPK signaling pathway (25). It is correlated with higher levels of proinflammatory cytokines and chemokines in bronchoalveolar lavage fluid in acute lung injury model (26), and anti-Histone H4 improves the survival of sepsis in mice (27). Thus, the higher level of Histone H4 in BD NETs might mediate aberrant macrophage activation, especially the induction of IL-8. Whether the higher level of Histone H4 in BD NETs mediated more Th1 cells differentiation remains further determined in our future studies. Besides, Le Joncour et al. report NETs promote thrombosis in BD patients (3). Since Histone H4 is cytotoxic for endothelial cells (27) and directly induces platelet aggregation (28), the higher level of Histone H4 in NETs might also contribute to the thrombosis in BD. Notably, other proteins in BD NETs might also play a role in regulating macrophages activation, which requires high-throughput methods to explore.

NETs also enrich nucleotides. 8-OHdG DNA is a marker of DNA oxidative damage and generated by ROS targeting at the C8 position of deoxyguanosine and adding hydroxyl radical (29). The more robust ROS production in BD neutrophils (4) might mediate the elevated 8-OHdG DNA in BD NETs. 8-OHdG DNA is a potent inducer of IL-6, TNF-α for PBMC (10). Lood C et al. show that 8-OHdG DNA is presented as a trace amount in NETs DNA and is a more potent inducer of proinflammatory cytokines than non-oxidized NETs DNA (10). Although we found a small increase in oxidized DNA in BD NETs compared with HC NETs, the proinflammatory effect of oxidized BD NETs DNA might be amplified by the overproduction of BD NETs. Given a higher level of oxidized NETs DNA potentially drive a stronger inflammation, the small increase in oxidized BD NETs DNA might play a role in BD inflammation. Besides, Gehrke N et al. report that the oxidized DNA, but not non-oxidized DNA, is resistant to exonuclease TREX1 degradation, accumulates in the cytoplasm and induces cGAS-STING-dependent type-I IFN (30). Thus, we proposed that BD neutrophils released NETs enriched with oxidized DNA, which might also promote proinflammatory cytokines in macrophages and drive the inflammatory in BD.

## Conclusions

In summary, excessive BD NETs promoted macrophages hyperactivation and the overproduction of proinflammatory cytokines and facilitated Th1 cells differentiation. A higher level of Histone H4 in BD NETs contributed to the overproduction of IL-8 by macrophages, and enriched oxidized DNA in BD NETs might also mediate macrophages hyperactivation.

## Data Availability Statement

The original contributions presented in the study are included in the article/**Supplementary Materials**. Further inquiries can be directed to the corresponding authors.

## Ethics Statement

The studies involving human participants were reviewed and approved by the institutional committee for the Protection of Human Subjects from PUMCH. The patients/participants provided their written informed consent to participate in this study.

## Author Contributions

WZ and HC conceptualized and designed the project and supervised the project. LL performed the experiments and wrote the manuscript. XY, JL, ZW, CL, and JS participated in the sample collection and clinical analysis. XY, JL, LS, and YL critically reviewed the manuscript and provided valuable input. All authors contributed to the article and approved the submitted version.

## Funding

This work was supported by the National Key Research and Development Program: “Precise Medical Research” [grant number 2016YFC0906201]; National Natural Science Foundation of China [grant number 81871299]; CAMS Innovation Fund for Medical Sciences [grant number 2016-I2M-1-013] [grant number2017-I2M-1-008].

## Conflict of Interest

The authors declare that the research was conducted in the absence of any commercial or financial relationships that could be construed as a potential conflict of interest.

## References

[1] Matsumura N Mizushima Y Leucocyte movement and colchicine treatment in Behcet’s disease. Lancet (1975) 2:813. 10.1016/s0140-6736(75)80031-6 78172

[2] Brinkmann V Reichard U Goosmann C Fauler B Uhlemann Y Weiss DS Neutrophil extracellular traps kill bacteria. Science (2004) 303:1532–5. 10.1126/science.1092385 15001782

[3] Le Joncour A Martos R Loyau S Lelay N Dossier A Cazes A Critical role of neutrophil extracellular traps (NETs) in patients with Behcet’s disease. Ann Rheum Dis (2019) 78:1274–82. 10.1136/annrheumdis-2018-214335 31147357

[4] Safi R Kallas R Bardawil T Mehanna CJ Abbas O Hamam R Neutrophils contribute to vasculitis by increased release of neutrophil extracellular traps in Behcet’s disease. J Dermatol Sci (2018) 92:143–50. 10.1016/j.jdermsci.2018.08.010 30237006

[5] Farrera C Fadeel B Macrophage clearance of neutrophil extracellular traps is a silent process. J Immunol (2013) 191:2647–56. 10.4049/jimmunol.1300436 23904163

[6] Mistry P Carmona-Rivera C Ombrello AK Hoffmann P Seto NL Jones A Dysregulated neutrophil responses and neutrophil extracellular trap formation and degradation in PAPA syndrome. Ann Rheum Dis (2018) 77:1825–33. 10.1136/annrheumdis-2018-213746 PMC6728908 30131320

[7] Barrera-Vargas A Gomez-Martin D Carmona-Rivera C Merayo-Chalico J Torres-Ruiz J Manna Z Differential ubiquitination in NETs regulates macrophage responses in systemic lupus erythematosus. Ann Rheum Dis (2018) 77:944–50. 10.1136/annrheumdis-2017-212617 PMC6560641 29588275

[8] Hu Q Shi H Zeng T Liu H Su Y Cheng X Increased neutrophil extracellular traps activate NLRP3 and inflammatory macrophages in adult-onset Still’s disease. Arthritis Res Ther (2019) 21:9. 10.1186/s13075-018-1800-z 30616678 PMC6323819

[9] Najmeh S Cools-Lartigue J Giannias B Spicer J Ferri LE Simplified Human Neutrophil Extracellular Traps (NETs) Isolation and Handling. J Vis Exp (2015) 98:e52687. 10.3791/52687 PMC4541576 25938591

[10] Lood C Blanco LP Purmalek MM Carmona-Rivera C De Ravin SS Smith CK Neutrophil extracellular traps enriched in oxidized mitochondrial DNA are interferogenic and contribute to lupus-like disease. Nat Med (2016) 22:146–53. 10.1038/nm.4027 PMC4742415 26779811

[11] Perazzio SF Soeiro-Pereira PV Dos SV de Brito MV Salu B Oliva M Soluble CD40L is associated with increased oxidative burst and neutrophil extracellular trap release in Behcet’s disease. Arthritis Res Ther (2017) 19:235. 10.1186/s13075-017-1443-5 29052524 PMC5649058

[12] Neves FS Spiller F Possible mechanisms of neutrophil activation in Behcet’s disease. Int Immunopharmacol (2013) 17:1206–10. 10.1016/j.intimp.2013.07.017 23973446

[13] Burdon PC Martin C Rankin SM The CXC chemokine MIP-2 stimulates neutrophil mobilization from the rat bone marrow in a CD49d-dependent manner. Blood (2005) 105:2543–8. 10.1182/blood-2004-08-3193 15542579

[14] Gur-Toy G Lenk N Yalcin B Aksaray S Alli N Serum interleukin-8 as a serologic marker of activity in Behcet’s disease. Int J Dermatol (2005) 44:657–60. 10.1111/j.1365-4632.2004.02184.x 16101867

[15] Dalghous AM Freysdottir J Fortune F Expression of cytokines, chemokines, and chemokine receptors in oral ulcers of patients with Behcet’s disease (BD) and recurrent aphthous stomatitis is Th1-associated, although Th2-association is also observed in patients with BD. Scand J Rheumatol (2006) 35:472–5. 10.1080/03009740600905380 17343257

[16] Markomichelakis N Delicha E Masselos S Fragiadaki K Kaklamanis P Sfikakis PP A single infliximab infusion vs corticosteroids for acute panuveitis attacks in Behcet’s disease: a comparative 4-week study. Rheumatol (Oxford) (2011) 50:593–7. 10.1093/rheumatology/keq366 21097877

[17] Kinoshita H Kunisaki R Yamamoto H Matsuda R Sasaki T Kimura H Efficacy of infliximab in patients with intestinal Behcet’s disease refractory to conventional medication. Intern Med (2013) 52:1855–62. 10.2169/internalmedicine.52.0589 23994973

[18] Salmaninejad A Zamani MR Shabgah AG Hosseini S Mollaei F Hosseini N Behcet’s disease: An immunogenetic perspective. J Cell Physiol (2019) 234:8055–74. 10.1002/jcp.27576 30341905

[19] Lim CH Adav SS Sze SK Choong YK Saravanan R Schmidtchen A Thrombin and Plasmin Alter the Proteome of Neutrophil Extracellular Traps. Front Immunol (2018) 9:1554. 10.3389/fimmu.2018.01554 30038618 PMC6046383

[20] Urban CF Ermert D Schmid M Abu-Abed U Goosmann C Nacken W Neutrophil extracellular traps contain calprotectin, a cytosolic protein complex involved in host defense against Candida albicans. PloS Pathog (2009) 5:e1000639. 10.1371/journal.ppat.1000639 19876394 PMC2763347

[21] O’Donoghue AJ Jin Y Knudsen GM Perera NC Jenne DE Murphy JE Global substrate profiling of proteases in human neutrophil extracellular traps reveals consensus motif predominantly contributed by elastase. PloS One (2013) 8:e75141. 10.1371/journal.pone.0075141 24073241 PMC3779220

[22] Chen R Kang R Fan XG Tang D Release and activity of histone in diseases. Cell Death Dis (2014) 5:e1370. 10.1038/cddis.2014.337 25118930 PMC4454312

[23] Devaney JM Greene CM Taggart CC Carroll TP O’Neill SJ McElvaney NG Neutrophil elastase up-regulates interleukin-8 via toll-like receptor 4. FEBS Lett (2003) 544:129–32. 10.1016/s0014-5793(03)00482-4 12782302

[24] Vogl T Tenbrock K Ludwig S Leukert N Ehrhardt C van Zoelen MA Mrp8 and Mrp14 are endogenous activators of Toll-like receptor 4, promoting lethal, endotoxin-induced shock. Nat Med (2007) 13:1042–9. 10.1038/nm1638 17767165

[25] Huang H Evankovich J Yan W Nace G Zhang L Ross M Endogenous histones function as alarmins in sterile inflammatory liver injury through Toll-like receptor 9 in mice. Hepatology (2011) 54:999–1008. 10.1002/hep.24501 21721026 PMC3213322

[26] Bosmann M Grailer JJ Ruemmler R Russkamp NF Zetoune FS Sarma JV Extracellular histones are essential effectors of C5aR- and C5L2-mediated tissue damage and inflammation in acute lung injury. FASEB J (2013) 27:5010–21. 10.1096/fj.13-236380 PMC3834784 23982144

[27] Xu J Zhang X Pelayo R Monestier M Ammollo CT Semeraro F Extracellular histones are major mediators of death in sepsis. Nat Med (2009) 15:1318–21. 10.1038/nm.2053 PMC2783754 19855397

[28] Xu J Zhang X Monestier M Esmon NL Esmon CT Extracellular histones are mediators of death through TLR2 and TLR4 in mouse fatal liver injury. J Immunol (2011) 187:2626–31. 10.4049/jimmunol.1003930 PMC3159755 21784973

[29] Bigagli E Lodovici M Circulating Oxidative Stress Biomarkers in Clinical Studies on Type 2 Diabetes and Its Complications. Oxid Med Cell LONGEV (2019) 2019:e5953685. 10.1155/2019/5953685 PMC6535859 31214280

[30] Gehrke N Mertens C Zillinger T Wenzel J Bald T Zahn S Oxidative damage of DNA confers resistance to cytosolic nuclease TREX1 degradation and potentiates STING-dependent immune sensing. Immunity (2013) 39:482–95. 10.1016/j.immuni.2013.08.004 23993650

