# Supplementary material for: Neutrophil Extracellular Traps Promote Aberrant Macrophages Activation in Behçet’s Disease
Source: Front Immunol. 2021 Feb 5;11:590622. doi: 10.3389/fimmu.2020.590622 (PMC7901995; doi:10.3389/fimmu.2020.590622)
Supplement: Supplementary file 1 [file Image_1.pdf]

Supplementary Material

Supplementary Figures

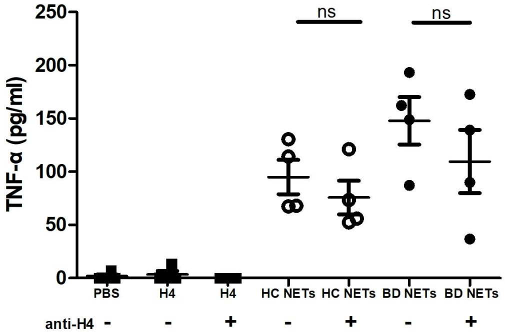

Supplementary Figure 1. Histone H4 in BD NETs didn't contribute to the overproduction of TNF-α by macrophages. HC NETs (n=4) and BD NETs (n=4) were pretreated with anti-Histone H4 antibody and stimulated macrophages for 24 hr. TNF-α production was measured using ELISA. Bars indicate mean value and Standard Deviation. Paired t-test for comparing the differences between before and after blocking Histone H4. BD, Behçet's disease; HC healthy controls; ns, not significant.

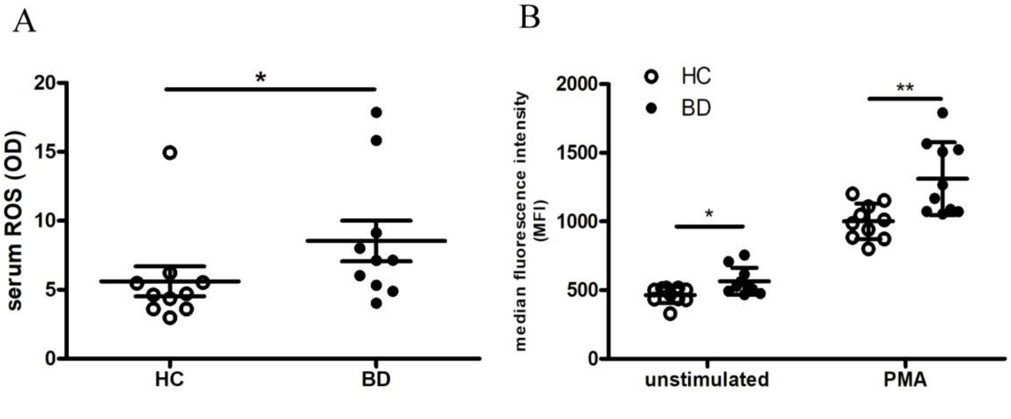

Supplementary Figure 2. The levels of ROS in BD serum and neutrophils were higher than those of HC. (A) Serum ROS from HC (n=10) and BD (n=10). (B) Unstimulated or 25nM PMA-stimulated HC (n=10) and BD (n=10) neutrophils were incubated for 10 min, and the median fluorescence intensity was measured by flow cytometry. Bars indicate mean value and Standard Deviation. Mann-Whitney U test for (A). Student's t-test for (B). * $p<0.05$, ** $p<0.01$. BD, Behçet's disease; HC healthy controls; ns, not significant.
