# Supplementary material for: Neutrophil Extracellular Traps Promote Aberrant Macrophages Activation in Behçet’s Disease
Source: Front Immunol. 2021 Feb 5;11:590622. doi: 10.3389/fimmu.2020.590622 (PMC7901995; doi:10.3389/fimmu.2020.590622)
Supplement: Supplementary file 2 [file Table_1.docx]

Supplementary Material

# Supplementary Table

Table 1 Clinical characteristics of BD patients and healthy controls.

|  | BD patients(n=38) | HC (n=36) |
| --- | --- | --- |
| Age (years, mean±standard deviation) | 35±10 | 37±9 |
| Sex(male, %) | 21 (55%) | 20 (56%) |
| Clinical Features (n, %) |  |  |
| Oral ulcers | 38 (100%) | - |
| Genital ulcers | 31 (82%) | - |
| Skin involvement | 27 (71%) | - |
| Vascular involvement | 13 (34%) | - |
| Arthritis | 6 (16%) | - |
| Gut involvement | 5 (13%) | - |
| Pathergy reaction | 4 (11%) | - |
| Neurological involvement | 2 (5%) | - |
| Uveitis | 2 (7%) | - |
| ESR (mm/1h), median (range) | 27 (2, 100) | - |
| CRP (mg/L), median (range) | 17 (0.4, 101.5) | - |
| BDCAF, median (range) | 2 (0, 3) | - |

BD, Behcet’s disease; HC, Healthy control; ESR, erythrocyte sedimentation rate; CRP, C-reactive protein; BDCAF, Behcet’s Disease current activity 2006 Form.
